# Viral pre-challenge increases central nervous system inflammation after intracranial interleukin-1β injection

**DOI:** 10.1186/s12974-014-0178-3

**Published:** 2014-10-17

**Authors:** Yvonne Couch, Andrew E Davis, Inês Sá-Pereira, Sandra J Campbell, Daniel C Anthony

**Affiliations:** Department of Pharmacology, University of Oxford, Mansfield Road, OX1 3QT Oxford, UK

**Keywords:** Pre-challenge, Interleukin-1β, Adenovirus, CNS injury, Inflammation, Rat

## Abstract

**Introduction:**

Systemic inflammation has been shown to significantly worsen the outcome of neurological disease. However, after acute injuries to the brain both pre- and post-conditioning with bacterial endotoxin has been shown to reduce leukocyte recruitment to the CNS. Here, we sought to determine whether viral pre-challenge would have an effect on the outcome of acute CNS inflammation that was distinct from endotoxin.

**Methods:**

Animals received a single intracranial microinjection of IL-1β in the presence or absence of a viral pre-challenge 24 hours prior to surgery. Liver and brain tissue were analysed for chemokine expression by qRT-PCR and leukocyte and monocyte infiltration 12 hours, 3 days and 7 days after the IL-1β injection.

**Results:**

Here, a single injection of adenovirus prior to IL-1β injection resulted in adhesion molecule expression, chemokine expression and the recruitment of neutrophils to the injured CNS in significantly higher numbers than in IL-1β injected animals. The distribution and persistence of leukocytes within the CNS was also greater after pre-challenge, with neutrophils being found in both the ipsilateral and contralateral hemispheres. Thus, despite the absence of virus within the CNS, the presence of virus within the periphery was sufficient to exacerbate CNS disease.

**Conclusions:**

These data suggest that the effect of a peripheral inflammatory challenge on the outcome of CNS injury or disease is not generic and will be highly dependent on the nature of the pathogen.

**Electronic supplementary material:**

The online version of this article (doi:10.1186/s12974-014-0178-3) contains supplementary material, which is available to authorized users.

## Introduction

Systemic infections are known to be associated with the exacerbation of central nervous system (CNS) pathologies such as multiple sclerosis (MS) [1], stroke [2], Parkinson’s and Alzheimer’s diseases [3-5]. Experimentally, there is also evidence to suggest that the combination of an infection model with, for example, murine prion disease [6], or with the middle cerebral artery occlusion model of ischemia [7], will exacerbate the CNS pathology. We have shown that bidirectional communication between the brain and the peripheral immune system revolves around the hepatic production of cytokines and chemokines. Moreover, that concomitant pathologies which are capable of eliciting an acute phase response (APR) have the potential to interfere with the immune response to acute brain injury [8,9]. An increased hepatic APR to brain injury and disease would suggest that any factors that exacerbate the systemic inflammatory response are likely to adversely affect disease outcome [1,10-12].

We have shown that the administration of individual exogenous APRs, such as CCL-2, will exacerbate a brain injury model. However, it is clear that the situation is considerably more complicated. Pre- and post-conditioning studies suggest that stimulating the immune system can confer a degree of tolerance to CNS injury. In animal models of stroke, a number of different strategies, including brief periods of ischemia, afford a degree of neuroprotection against a subsequent insult [13-15]. Surprisingly, the systemic administration of bacterial lipopolysaccharides (LPS) has been reported to confer neuroprotection from a subsequent ischemic challenge [16,17]. While the mechanism of protective pre-conditioning strategies might include the activation of the hypothalamic-pituitary-adrenal axis, or a form of tachyphylaxis, it is harder to explain how protective post-conditioning is achieved. We have shown that pre-conditioning and post-conditioning using LPS in models of both brain and spinal cord injury using intracranial IL-1β and spinal cord compression, shows reduced CNS immune activation and improved outcome [18]. In the latter we found it was possible to inhibit the recruitment of leukocytes into the spinal cord after injury using an intravenous injection of LPS before, and, importantly, after, the compression injury. The peripheral administration of IL-1 [18] did not have the same effect, suggesting an important role for toll-like receptor (TLR) signalling pathways. However, hitherto, it remains unclear whether or not other TLR pathway activating pathogens, such as viruses, might have similar LPS-like pre-conditioning effects. Currently, approximately one third of MS relapses are preceded by a viral infection, and the rate of relapse appears to be two to three fold higher following viral infection [19-23]. Thus, it might be predicted that viral infections should cause exacerbation of CNS lesions. However, the same association is true for bacterial infections where respiratory and urinary tracts caused by bacteria also appear to trigger relapse in 20 to 30% of MS patients [22,24]. Activation of innate immunity by adenoviruses has been reported to occur through both TLR-dependent and TLR-independent pathways, but adenoviruses seem to activate the innate immune system principally via TLR signalling pathways [25]. Given how widespread adenovirus use has become, here we sought to investigate how pre-challenge with a dsDNA (double stranded DNA) virus might affect the pathogenesis of an acute sterile CNS inflammatory lesion.

## Subjects and methods

### Animals

Adult 8-week-old (200 g), male Wistar rats (Harlan, Bicester, UK) were housed in specific pathogen-free (SPF) facilities under a standard light/dark cycle with food and water *ad libitum*. In each experiment, at least three animals were used per treatment group and per time point. All procedures were carried out in accordance with the UK Animals (Scientific Procedures) Act, 1986 and with approval of local and European ethical committees, and all reasonable efforts were made to minimise discomfort. Animals were randomly assigned to experimental, groups as per the ARRIVE guidelines [26].

### Pre-challenge and intrastriatal injection of IL-1β

Animals were anaesthetised with 2 to 3% isoflurane in oxygen. All surgical procedures were performed under an operating microscope (Wild M650, Leica, Milton Keynes, UK). Stereotaxic surgery was performed as described previously [27]. Briefly, the head of the anaesthetised animal was shaved and held in a stereotaxic frame (Kopf Instruments, Tajunga, California). The skull was then exposed with a midline sagittal incision and dental burr drill was used to make a small hole for the injection (co-ordinates from bregma: A/Pr +1.2 mm, M/L +3.0 mm, D/V −4.5 mm). One microlitre of rrIL-1β (recombinant rodent-IL-1β; National Institute for Biological Standards and Control (NISBC), Potters Bar, UK; 1 μg/μl) or vehicle (0.9% saline) was microinjected into the left striatum through a pulled glass microcapillary needle (tip <50 μm), as our standard laboratory model of brain inflammation [28]. The cytokine was delivered over a period of 2 minutes and the micropipette was then left in place for a further 5 minutes to allow the injected cytokine to diffuse away from the injection site and prevent reflux up the injection tract. Local anaesthetics were applied to wound sites prior to recovery. Body temperature was maintained on a heated blanket throughout the period of anaesthesia and the animals were allowed to recover in a heated chamber thereafter. For pre-challenge studies, animals were briefly anaesthetised using 2% isoflurane via a nose-cone and injected intravenously with either a luciferase-expressing adenovirus (*AdLuc* kindly provided by Prof. Len Seymour, Oxford) or vehicle (0.9% saline) 24 hours prior to stereotaxic surgery. Virus was injected intravenously (iv) at a concentration of 5 × 10^9^ pfu (plaque-forming unit)/animal.

### Tissue preparation

Animals were surgically anaesthetised and blood was collected via cardiac puncture into EDTA-coated tubes (Teklab, Durham, UK), or into serum separators (Southern Syringe Services Ltd, London, UK). Animals were then transcardially perfused with 0.9% saline containing 5,000 U/l heparin followed by a periodate lysine paraformaldehyde solution (PLP: 2% paraformaldehyde, lysine, periodate and 0.05% glutaraldehyde). Tissues were removed, post-fixed in PLP for 4 hours and further fixed in 30% sucrose for >12 hours. Ten-micron-thick sections were cut on a cryostat (Leica, Bucks, UK) and mounted on gelatine-coated slides. One-microlitre volumes of whole blood were taken from the EDTA coated tubes and films were produced on clean glass slides. Blood films were allowed to dry at room temperature for 5 minutes before being stored at −20°C.

### Immunohistochemistry

Immunohistochemistry was performed as described previously [29]. Briefly, tissue sections were quenched for endogenous peroxidase activity in alcoholic hydrogen peroxide (H_2_O_2_) solution (0.03% H_2_O_2_ in methanol). Using the Shandon-Sequenza system, non-specific Fc-dependent binding was blocked with 10% normal serum (from the species in which the secondary antibody was raised) and primary antibody incubation took place. These included anti-neutrophil serum (HB-199, made in house; 1:10,000, 2 hours at room temperature); anti-ED-1 (mouse monoclonal, AbD Serotec, Oxford, UK; 1:200 overnight at room temperature); anti-ICAM-1 (mouse monoclonal, BD Pharmingen, Oxford, UK; 1:50, 2 hours at room temperature); anti-luciferase (goat polyclonal, Promega, Southampton, UK; 1:1,000 overnight at room temperature) and anti-CD11b (OX-42 clone, Serotec, Oxford, UK; 1:200 overnight at room temperature). Sections were then incubated with the appropriate biotinylated secondary antibody (Vector Laboratories, Peterborough, UK) before an avidin-biotin-peroxidase solution (Vectastain Elite ABC, Vector Laboratories, Peterborough, UK) was added. Peroxidase labelling was visualised by exposure to the chromogen diaminobenzidine hydrochloride (0.5 μg/ml) in 0.1 M phosphate buffer (0.05% H_2_O_2_) for varying times until optimal contrast was achieved. To control for endogenous biotin present in the liver, a separate avidin-biotin blocking procedure using an avidin-biotin blocking kit (Vector Laboratories, Peterborough, UK) was employed. For CNS tissue, samples were taken serially through the injection site. For blood counts, smears of 1 μl of blood were taken in triplicate and stained as above. For each analysis, four representative fields were chosen for quantitation and the average number of positive cells was calculated and expressed as number of cells per mm^2^. Tissue sections were counterstained with cresyl violet and immuno-positive labelling was quantitated only when associated with a cell nucleus. Spatial maps of positive immunoreactivity were created using plate 15 from the rat brain atlas [29]. Specifically, the sections were viewed at 1.6 magnification using a microscope attached to a camera lucida; positive staining was dotted onto white paper using a black marker pen. Images were scanned and superimposed onto plate 15 of the rat brain atlas to form spatial maps of overall immunoreactivity. All immunohistochemistry was performed blind, as per ARRIVE guidelines [26].

### Quantitative RT-PCR

One cubic millimetre punches of striatal tissue at the level of the injection were removed for RNA extraction. RT-PCR assays were performed as previously described [30]. Samples were run against absolute copy number standard curves generated from serially diluted cDNA from LPS-challenged rat liver. Primer and probe sets for rat IL-1β, CXCL-1, CCL-2, CXCL-10, CCL-3 (MIP-1α), and CCL-4 (MIP-1β) were designed using the Roche universal probe library assay design centre (Roche.com). Samples were analysed using a Roche Light Cycler 480® (Roche Diagnostics, Welwyn Garden City, UK) and all reagents were used according to manufacturer's instructions. Briefly, gene-specific primers were combined with a FAM/TAMRA labelled hybridisation probe (for sequences see Additional file 1: Table S1). PCR was run according to standard conditions [31]. Analysis was performed using the Pfaffle method, taking the standard curve to determine reaction efficiency followed by a comparative-cycle-threshold method. Results are expressed as absolute copy number.

### Statistics

Data are presented as mean ± standard error of the mean (SEM) for a minimum of three animals at each time point. Data were analysed using Student's *t*-test and two-way ANOVA with Bonferroni-Dunn *post hoc* testing for multiple groups where appropriate. Results were considered statistically significant when *P* <0.05 as compared to vehicle-treated or naïve controls where appropriate.

## Results

### Chemokine expression in the CNS is selectively affected by viral pre-challenge

**Figure 1:**
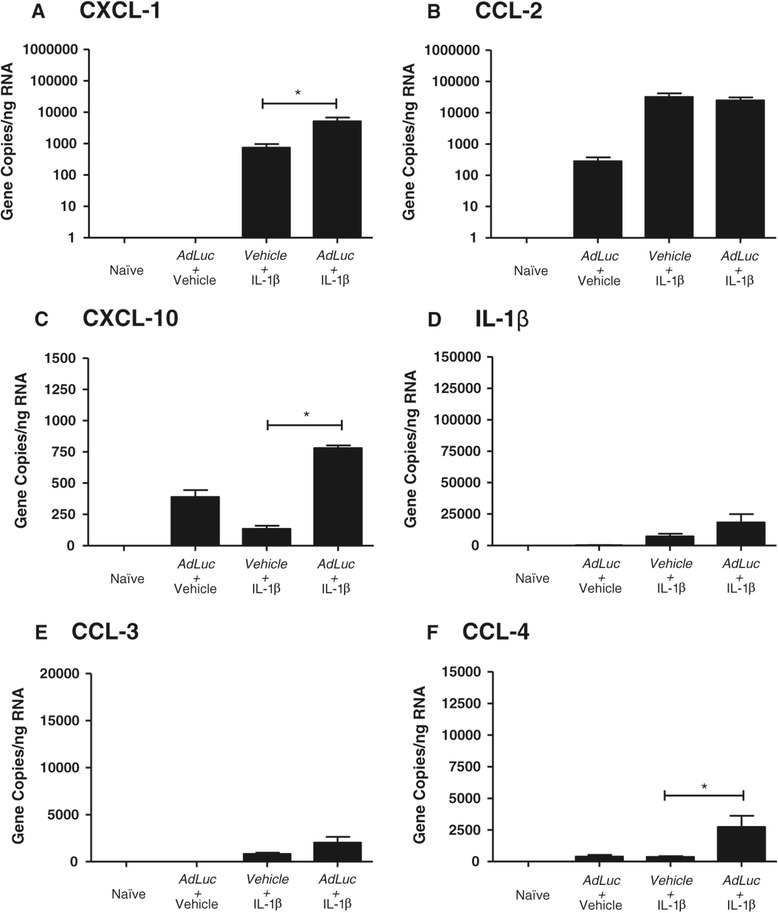
**Viral pre**-**challenge selectively increases chemokine mRNA expression in the brain 3 days following the microinjection of IL**-**1β.** The microinjection of IL-1β into the brain caused changes in absolute gene copies of mRNA for **(A)** CXCL-1; **(B)** CCL-2; **(C)** CXCL-10; **(D)** IL-1β; **(E)** CCL-3 and **(F)** CCL-4. Intravenous injections are indicated by italic text. Results are expressed as mean ± SEM (n =3), *P <0.05.

In keeping with previous observations, the microinjection of IL-1β into the brain caused an up-regulation of cytokines and chemokines known to be involved in leukocyte migration and recruitment (CXCL-1, IL-1β, and CCL-3) at 12 hours post-injection (Additional file 2: Figure S1). The microinjection of IL-1β did not induce the expression of TNF in the brain (data not shown), which, while surprising, supports previously reported findings [32]. While cytokine and chemokine expression profiles were examined at all time points, significant differences in expression were found between vehicle/IL-1β and *AdLuc*/IL-1β at 3 days post-injection (Figure 1). At this time point the microinjection of IL-1β into the brain had caused an elevation in mRNA for CXCL-1, CCL-2, IL-1β and CCL-3 relative to naïve levels (Figure 1). However, the presence of the viral load caused an up-regulation in CXCL-1, CCL-4 and CXCL-10 production following the microinjection of IL-1β when compared to vehicle/IL-1β injection alone (Figure 1). It was of note that the microinjection of vehicle, in the presence of *AdLuc* also induced an up-regulation in CXCL-10 production in the brain. By 7 days, only CCL-2 and CXCL-1 remained elevated in all IL-1β injected animals (with virus or without) compared to naïve or *AdLuc*/saline-injected animals (Additional file 3: Figure S2); at this time none of the other chemokine mRNA species measured in the CNS were elevated above naïve levels. It is important to note that immunohistochemistry for luciferase revealed the presence of luciferase protein in the liver, but not in the brain of the challenged animals (Additional file 4: Figure S3).

#### *Viral pre*-*challenge increases the acute mobilisation of neutrophils to the blood and to the liver in response to intrastriatal IL*-*1β injection*

**Figure 2:**
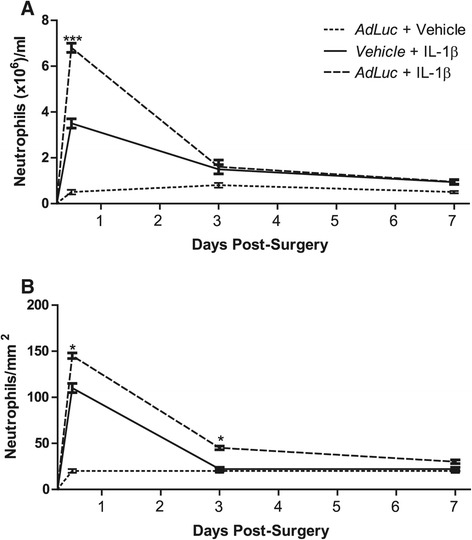
**Viral pre**-**challenge increases the acute mobilisation of neutrophils in response to the microinjection of IL**-**1β into the brain.** Neutrophils immunohistochemically assessed in **(A)** whole blood and **(B)** liver 12 hours, 3 days or 7 days following IL-1β challenge to the brain in the presence of intravenous vehicle (solid line) or *AdLuc* (5 × 10^9^ CFU - dashed line). Dotted line represents animals microinjected with saline into the brain, in the presence of intravenous *AdLuc*. Intravenous injections are indicated by italic text. Results are expressed as mean ± SEM (n =3), ***P <0.001. CFU, colony-forming unit.

The microinjection of IL-1β into the brain induced a rapid leukocytosis. Levels of circulating neutrophils rose rapidly, peaked at 12 hours, and had fallen by 3 days, although at this time they were still slightly above baseline. Levels did not change between 3 days and 7 days. When the microinjection of IL-1β was preceded by intravenous challenge with *AdLuc*, the number circulating neutrophils was markedly increased at 12 hours (*P* <0.001). By 3 days, levels had fallen again and were comparable to the vehicle/IL-1β group at this time. Neutrophil numbers again remained stable between 3 days and 7 days, marginally above baseline. The microinjection of vehicle into the brain, in the presence of intravenous *AdLuc*, did not induce the mobilisation of neutrophils to the blood above baseline levels (Figure 2A). Twelve hours after the microinjection of IL-1β, preceded by challenge with *AdLuc*, the numbers of neutrophils (*P* <0.05) present in the liver were significantly increased (*P* <0.05, Figure 2B), which is indicative of an altered APR. That neutrophils were still present in elevated numbers at 3 days (*P* <0.05) in the presence of *AdLuc* (*P* <0.001, Figure 2B) is also indicative of a prolonged APR. When animals were microinjected with vehicle into the brain, in the presence of intravenous *AdLuc*, no elevation of neutrophil numbers above baseline were observed in the liver (Figure 2B). Despite the observed increases in leukocyte number in the livers of animals receiving the combined challenge (*AdLuc*/IL-1β), no increase in chemokine expression caused by the presence of the virus was observed. However, it is of note that CCL-3 and CCL-4 mRNA was significantly up-regulated in all the animals that received the virus (Additional file 5: Figure S4).

### Adhesion molecule expression is increased in the CNS in response to viral pre-challenge

**Figure 3:**
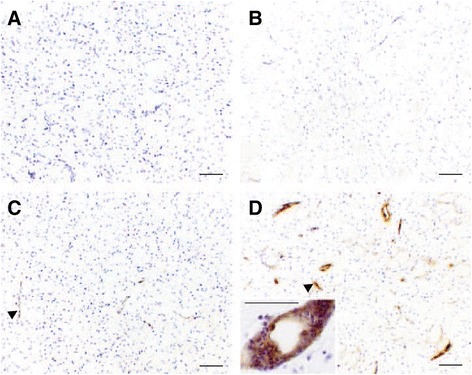
**Viral pre**-**challenge increases the expression of ICAM**-**1 in the brain.** Baseline ICAM-1 expression **(A)** did not differ significantly from ICAM-1 expression after the microinjection of vehicle, in the presence of intravenous *AdLuc* **(B)**. Intrastriatal IL-1β induced a generalised up-regulation of ICAM-1 expression, which was most evident around vessels actively involved in recruitment of leukocytes (**C**, arrowhead). In the brains of animals microinjected with IL-1β, in the presence of intravenous *AdLuc* (5 × 10^9^ CFU), this up-regulation was more notable still **(D)**. Note the two distinct subtypes of vessels; smaller vessels not actively involved in recruitment (arrowhead) and larger vessels showing increased ICAM-1 positivity, which were the ones where cuffs of neutrophils could be seen (inset). Counterstain is cresyl violet. Scale bars represent 50 μm. CFU, colony-forming unit.

The microinjection of vehicle, in the presence of intravenous *AdLuc*, did not cause any observable changes in the expression of ICAM-1 relative to baseline levels observed in naïve animals (Figure 3A and B). As expected, the microinjection of IL-1β into the brain induced widespread up-regulation of ICAM-1 expression (Figure 3C) that was most prominent around vessels actively involved in recruitment of leukocytes to the injected striatum. Some staining was also evident, to a lesser extent, in the cortex in the ipsilateral hemisphere. When animals were microinjected with IL-1β into the brain, in the presence of intravenous *AdLuc*, the up-regulation in ICAM-1 expression appeared more widespread (Figure 3D). Again, the strongest ICAM-1 positivity was observed in the ipsilateral hemisphere, with further up-regulation above naïve in other areas actively involved in recruitment. The most notable observation was that in the striatum pre-conditioned IL-1β animals there appeared to be two populations of ICAM-1+ vessels. There were small vessels positive for ICAM-1, but not actively involved in recruitment (arrowhead, Figure 3D), and those heavily positive for ICAM-1, and cuffed by large numbers of neutrophils (arrow, and inset, Figure 3D).

### Leukocyte recruitment to the IL-1β-challenged brain is enhanced by viral pre-challenge

**Figure 4:**
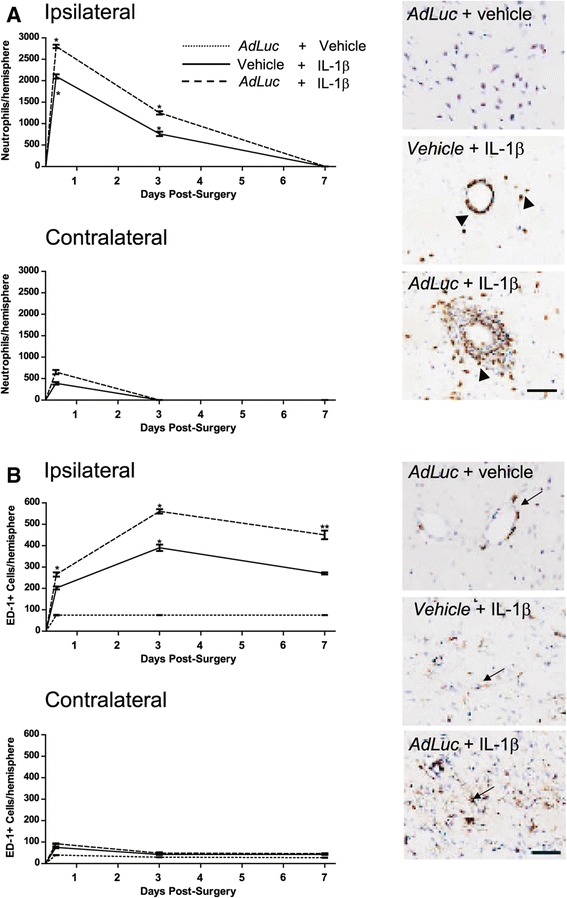
**Viral pre**-**challenge increases the recruitment of neutrophils and ED**-**1**-**positive macrophages to the IL**-**1β-challenged brain.** Graphs show numbers of neutrophils **(A)** and ED-1-positive cells **(B)** in the ipsilateral and contralateral hemisphere at 12 hours, 3 days and 7 days after the microinjection of IL-1β, in the presence of intravenous vehicle (solid lines) or *AdLuc* (5 × 10^9^ CFU - dashed lines). Animals microinjected with vehicle into the brain, in the presence of intravenous *AdLuc* are shown by dotted line (not visible in **(A)** due to low constitutive neutrophil numbers). Immuno-positive neutrophils (**A** - arrowheads) and ED-1-positive macrophages (**B** - arrows) are shown in brown. Intravenous injections are indicated by italic text. Counterstain is cresyl violet. Scale bar represents 50 μm. Results are expressed as mean ± SEM (n =3). Statistical significance is indicated by **P <0.01, ***P <0.001 respectively. CFU, colony-forming unit.

The microinjection of IL-1β into the brain in the presence of intravenous vehicle resulted in elevated numbers of neutrophils present in the ipsilateral hemisphere from 12 hours post-injection (Figure 1A). By 3 days, the number of neutrophils had dropped to approximately 35% of those seen at 12 hours, and by 7 days had reduced to baseline. The presence of intravenous *AdLuc* administered 24 hours prior to the microinjection of IL-1β caused an elevation in the numbers of neutrophils observed in the injected hemisphere at 12 hours as compared to vehicle (*P* <0.01), which persisted to 3 days post-injection. At this time, neutrophils were presents in large cuffs around vessels in the injected striatum in animals receiving *AdLuc* that was not evident in other groups (Figure 4A, arrowheads). By 7 days, neutrophil numbers were for the most part back to baseline, although slightly more were observed in animals that received *AdLuc*, than the IL-1β alone group. In animals microinjected with vehicle into the brain, in the presence of intravenous *AdLuc*, no measurable numbers of neutrophils were observed in the brain at any time point studied. The microinjection of IL-1β into the brain in the presence of intravenous vehicle also resulted in some recruitment of neutrophils to the contralateral hemisphere 12 hours after the striatal injection, although at a much lower level than in the injected hemisphere (Figure 4A). These were mainly observable in the cortex, and very few were present in the striatum. By 3 days, the numbers of neutrophils had returned to baseline levels. The presence of *AdLuc* administered 24 hours prior to the microinjection of IL-1β caused an elevation in the numbers of neutrophils observed in the contralateral hemisphere at 12 hours, but by 3 days the numbers of neutrophils were again back to baseline.

The microinjection of IL-1β into the brain, in the presence of intravenous vehicle, also elevated the number of ED-1-positive cells present in the ipsilateral hemisphere from 12 hours to 7 days (Figure 4B). The number peaked at 3 days, but remained well above baseline until day 7. The presence of intravenous *AdLuc* administered 24 hours prior to the microinjection of IL-1β caused a further significant elevation in the numbers of ED-1-positive cells all time points. In the contralateral hemisphere, there was a small increase in the number of ED1-positive cells at 12 hours (*P* <0.05). By 3 days, the number of ED1 cells was slighted elevated over baseline, but similar in all the treatment groups.

### Viral pre-challenge alters the temporal profile of leukocyte recruitment to the IL-1β-challenged brain

**Figure 5:**
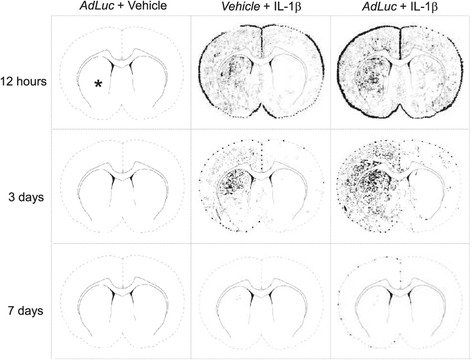
**Viral challenge increases temporal pattern of recruitment of neutrophils to the inflamed central nervous system (CNS).** Illustrations show schematic representations of the pattern of neutrophil recruitment to the challenged brain (* represents the microinjection site in the brain for all animals and time points). The microinjection of vehicle, in the presence of intravenous *AdLuc* (5 × 10^9^ CFU) did not cause recruitment of neutrophils to the brain at any time point (left column). The microinjection of IL-1β in the presence of intravenous vehicle (central column) caused bilateral meningitis, and the recruitment of neutrophils to the injected hemisphere in large numbers at 12 hours. By 3 days, the area over which neutrophil recruitment was occurring, and the numbers recruited were decreased. By 7 days, only isolated cells were observable. When the microinjection of IL-1β was combined with intravenous challenge with *AdLuc* (right column), neutrophil recruitment was widespread, covering both hemispheres at 12 hours post-injection, with significantly more cells recruited in the contralateral hemisphere than after IL-1β/vehicle. By 3 days, the area over which neutrophil recruitment was occurring was reduced, although again greater than in animals receiving intravenous vehicle. By 7 days, recruitment was near basal levels, although some meningitis was still evident. Intravenous injections are indicated by italic text. CFU, colony-forming unit.

In order to better represent the pattern of recruited leukocytes into the CNS, spatial maps were created to show the patterns and density of cellular recruitment over time. When animals were microinjected with vehicle, in the presence of intravenous *AdLuc*, no neutrophils were observed in the brain at any time point (Figure 5, left column). The microinjection of IL-1β in the presence of intravenous vehicle caused large numbers of neutrophils to be observed in the ipsilateral striatum and cortex at 12 hours (Figure 5, middle column). Limited numbers of neutrophils were observed in the contralateral cortex, and bilateral meningitis was present. By 3 days, significant numbers of neutrophils remained present in the injected striatum, but there was much less recruitment occurring in the cortex, and only isolated cells were observed in the contralateral hemisphere. At this time point, the meningitis was dramatically reduced in both spread and magnitude, being largely ipsilateral in nature. By 7 days, only isolated cells were observable in the injected striatum, the rest of the brain resembled naïve tissue. When the microinjection of IL-1β was combined with intravenous administration of *AdLuc* 24 hours beforehand, the numbers of neutrophils observed in the brain at 12 hours post-IL-1β was significantly increased (Figure 5, right column). Within the injected hemisphere, there were no dramatic changes in the manner of recruitment observed, merely that the vessels actively engaged in recruitment was spread over a larger area, contributing to the increase in numbers. The most striking changes were observed in the contralateral hemisphere, where the area in which active recruitment was occurring was much more widespread within the cortex, and to a lesser extent, within the striatum. By 3 days, a similar pattern was observed to that seen in animals injected with IL-β alone, but with some spatial differences. The area over which recruitment was occurring in the injected hemisphere was much larger, predominantly in the cortex. Furthermore, in the injected striatum, large numbers of neutrophils were cuffed around vessels, five to seven cells thick, indicating that the process of diapedesis was occurring to a much greater extent than in the IL-1β/vehicle group. By 7 days, the differences between the groups were less defined. In the group administered *AdLuc* there were marginally elevated numbers of neutrophils in the injected striatum, and there was still evidence of meningitis, although the majority of the section was devoid of neutrophils.

## Discussion

In contrast to our previous pre-conditioning studies using LPS [18], pre-challenge with the *AdLuc* adenovirus caused an extended CNS inflammatory response that was characterised by increased leukocyte recruitment lasting until 7 days post-injection, and this response was accompanied by an increase in adhesion molecule, cytokine, and chemokine expression within the ipsilateral hemisphere. These data suggest that the anti-inflammatory effect of the conditioning stimulus seen previously appears to be restricted to endotoxin-specific pathways and suggests that the systemic administration of *AdLuc* is likely to have an adverse effect on the outcome of co-morbid CNS inflammatory disease.

The ability of pre-conditioning stimuli to abrogate the deleterious effects of a systemic inflammatory response on the outcome of CNS injury is well documented. However, there are obvious shortcomings in terms of implementing protective pre-conditioning strategies, and, not least, among these there is the absence of a complete mechanistic understanding. Here, we have shown that the outcome of pre-conditioning strategy is highly dependent on the nature of the pre-conditioning challenge. As in previously studies [33], we found that *AdLuc* administered iv is not detectable in the brain (Additional file 4: Figure S3). It was considered important to show that the injection of virus did not result in any infection of CNS cell populations, which might provoke a local immune response and confound results. The peripheral iv injection of a replication deficient adenovirus expressing the NF-κB super-repressor or luciferase does not result in the expression of transcript in the brain [33] and here we show that the luciferase protein is only expressed in the liver. Others have shown that adenoviruses administered intravenously preferentially target the liver [34,35]. We found that this targeting of the liver resulted in a marked elevation in the production of mRNA for the related macrophage chemokines CCL-3 and CCL-4 in the liver (Additional file 5: Figure S4), irrespective of the challenge to the CNS. It should be noted that at 24 hours the luciferase reporter gene expression would not be expected to generate an immunological response *per se* and it is reasonable to assume that the chemokine response can be attributed to the vector. The host response to infection with adenoviral vectors (*AdLuc*) is known to result in the rapid activation of innate immunity, which stimulates inflammatory antiviral host defenses [36]. CCL-3 and CCL-4 are known to be involved in responses to certain viral infections [37], but, in addition to macrophage activation, they can drive cell proliferation and may encourage viral propagation such as in Epstein-Barr B-cell infection [38].

In the brain, the microinjection of IL-1β resulted in the recruitment of neutrophils and ED-1-positive macrophages to the ipsilateral hemisphere. Based on observations from our earlier LPS studies [18], this result was initially surprising. However, systemic infection is known to exacerbate inflammatory responses in the brain in many contexts [39,40] and the result with endotoxin was the more surprising. This provides a potentially useful tool to discover the mechanisms that lead to the viral exacerbation of CNS lesions, such as those occurring in MS, where epidemiological evidence has long suggested a role for virus infection in the initiation and/or exacerbation of the disease [41,42]. Theiler's murine encephalomyelitis virus (TMEV)-induced demyelinating disease has been used as a model for MS. TMEV-infected mice develop a demyelinating disease, which, eventually, is associated with development of myelin-specific T-cell responses. Viruses have been suggested to initiate autoimmune disease through bystander activation of immune cells or through bystander damage to tissue during infection. In this model, administration of antiserum to IFN-beta or poly(I:C), a TLR3 agonist mimicking a dsRNA viral infection, leads to more severe demyelinating disease by promoting ‘virus-like’ activity, specifically the recruitment and activation of ‘bystander’ immune cells within the tissue such as microglia and astrocytes [43]. It is interesting to note that this TLR3 pathway, activated by poly(I:C), also exacerbates CNS inflammation in a similar manner to our *AdLuc* TLR9 (and likely others) agonist. Others have also used poly(I:C) to investigate how systemic infection might contribute to the later development of Alzheimer disease and have shown the development of Alzheimer’s-like pathology following poly(I:C) exposure prenatally [44].

The observation that *AdLuc* pre-challenge increased the numbers of neutrophils present in the IL-1β-challenged brain for as long as 3 days following surgery is striking. Whilst limited infiltration of neutrophils may have a protective role following traumatic injury, elevated neutrophil numbers are also known to have damaging implications upon ischemic lesions in the brain, and furthermore, recent studies have implied a causative role for neutrophils in cortical injury [45]. Whilst the current study does not examine ischemic lesions, the implications for increased neutrophil recruitment over time are similar. With this in mind, we were interested to discover if there were any ongoing mechanisms in the brain, which could account for the increased cellular recruitment observed following *AdLuc* challenge. Elevated expression of ICAM-1 was found in animals microinjected with IL-1β, and further increased by the presence of *AdLuc*. Constitutive expression of ICAM-1 in the brain is known to be low, and the administration of *AdLuc* in conjunction with the microinjection of vehicle did not up-regulate expression in the brain. This suggests that the virus does not increase adhesion molecule expression *per se*, but it appears that the effects of virus and the microinjection of IL-1β act synergistically to enhance endothelial signals for recruitment of leukocytes. We have previously shown that blocking adhesion molecule expression significantly reduces neutrophil infiltration into the brain after an IL-1β injection [46] and suggest a similar mechanism may be tested in this system in order to determine the degree to which these molecules influence neutrophil trafficking across the endothelium.

Finally, the elevated ED-1-positive macrophage numbers in the brain following the microinjection of IL-1β was also an unusual observation. These effects were still evident 7 days following the IL-1β microinjection and the numbers were larger than we would ordinarily have expected considering that we understand leukocyte recruitment to be restricted primarily to neutrophils following the microinjection of IL-1β [47]. When the profile of chemokine expression in the brain was examined following IL-1β challenge, it was evident that the presence of *AdLuc* caused an up-regulation in the local production of not just CXCL-1, which we would expect to account for the elevated neutrophil numbers. Indeed, elevations in CCL-2 are known to mobilise monocytes to the injured CNS [31], but we also observed elevations in the production of CCL-3, CCL-4 and CXCL-10. CCL-3 and CCL-4 are known to be produced by glial cells within the CNS and have been shown to mobilise leukocytes to sites of injury within the CNS [48]. It seems feasible that the elevation in beta-chemokine production is responsible for the increased numbers of ED-1-positive cells observed. However, while astrocytes do produce chemokine it is important to note that macrophages/microglia also produce beta-chemokine and the increased levels of expression may reflect a downstream consequence of the increased number of ED-1 cells present following the viral challenge. For example, IL-1β induces CXCL-10 in astrocytes in culture [49], but it is also expressed by macrophages and has been shown to exacerbate experimental autoimmune encephalomyelitis [50]. CXCL-10 is reported to have a diverse set of effects in the CNS; it seems to be important for the clearance of virus within the CNS [51] but also induces significant free radical production and apoptosis [52].

In summary, we have shown that a systemic viral challenge causes increased recruitment of leukocytes to the site of IL-1β microinjection in the brain, and also to the liver. In comparison to our previous work with LPS [18], these data suggest that viral pre-challenge has deleterious effects on the outcome of CNS injury. When considered in context, viral infection should be considered a significant risk factor for worsened outcome following injuries to the CNS, and may also contribute to multi-organ dysfunction.

## Additional files

Additional file 1: Table S1. Primer and probe sequences for real-time polymerase chain reaction. Additional file 2: Figure S1. Viral pre-conditioning selectively increases chemokine expression in the brain 12 hours following the microinjection of IL-1β. The microinjection of IL-1β into the brain caused changes in absolute gene copies of mRNA for (A) CXCL-1; (B) CCL-2; (C) CXCL-10; (D) IL-1β; (E) CCL-3 and (F) CCL-4. Intravenous injections are indicated by italic text. Results are expressed as mean ± SEM (n =3), **P* <0.05. Additional file 3: Figure S2. Viral pre-conditioning selectively increases chemokine expression in the brain 7 days following the microinjection of IL-1β. The microinjection of IL-1β into the brain caused changes in absolute gene copies of mRNA for (A) CXCL-1; (B) CCL-2; (C) CXCL-10; (D) IL-1β; (E) CCL-3 and (F) CCL-4. Intravenous injections are indicated by italic text. Results are expressed as mean ± SEM (n =3), **P* < 0.05. Additional file 4: Figure S3.
*AdLuc* administered intravenously does not enter the brain. Micrographs illustrate representative sections of (A) brain, and (B) liver of animals administered *AdLuc* intravenously. In all treatment groups receiving intravenous *AdLuc*, no immuno-labelling for luciferase was observed in the brain at any time point, despite the microinjection of vehicle or IL-1β into the brain, but was clearly observed in the liver (B) of the same animals. Immuno-positivity for luciferase shown in brown (arrows). Scale bars represent 50 μm. Additional file 5: Figure S4. Viral pre-conditioning causes selective up-regulation of macrophage-specific chemokines in the liver, irrespective of the challenge to the brain. The microinjection of IL-1β into the brain did not alter naïve chemokine mRNA levels in the liver 3 days later. A pre-challenge with *AdLuc* caused significant increases in both (A) CCL-3 and (B) CCL-4, irrespective of the challenge into the brain. Intravenous injections are indicated by italic text. Results are expressed as mean ± SEM (n =3), **P* <0.05.

## Abbreviations

CNS: central nervous system
LPS: lipopolysaccharide
CFU: colony forming unit
IL-1β: interleukin-1β
CXCL: C-X-C chemokine ligand
CCL: C-C chemokine ligand
MS: multiple sclerosis
TMEV: Theiler’s murine encephalomyelitis virus
IFN: interferon
RT-PCR: real-time polymerase chain reaction
RNA: ribonucleic acid
mRNA: messenger ribonucleic acid
cDNA: complementary deoxyribonucleic acid
MIP: macrophage inflammatory protein
APR: acute phase response
