# Supplementary material for: Viral pre-challenge increases central nervous system inflammation after intracranial interleukin-1β injection
Source: J Neuroinflammation. 2014 Oct 17;11:178. doi: 10.1186/s12974-014-0178-3 (PMC4201684; doi:10.1186/s12974-014-0178-3)
Supplement: Additional file 1: Table S1. — Primer and probe sequences for real-time polymerase chain reaction. [file 12974_2014_178_MOESM1_ESM.pdf]

Marker	Accession Number	Forward Primer	Reverse Primer	Probe	Amplicon Size
IL-1β	NM031512.1	TGTGATGAAAGACGGCACA	CTTCTTCTTTGGGTATTGTTTGG	Roche probe 78	70nt
CXCL-1	NM030845.1	CACACTCCAACAGAGCACCA	TGACAGCGCAGCTCATTG	Roche probe 83	120nt
CXCL-10	NM139089	AAAGAGGGGAGCAGGAAGAA	CTCTGACTTCAACCAGCATACG	Roche probe 41	66nt
CCL-2	NM031530	AGCATCCACGTGCTGTCTC	GATCATCTTGCCAGTGAATGAG	Roche probe 62	78nt
CCL-3 (MIP-1α)	NM013025.2	CTGGAACGAAGTCTTCTCAGC	GAATTTGCCGTCCATAGGAG	Roche probe 40	77nt
CCL-4 (MIP-1β)	NM053858.1	GAGACCAGCAGCCTTTGC	CACAGATTTGCCTGCCTTTT	Roche probe 20	67nt
