# Supplementary material for: Viral pre-challenge increases central nervous system inflammation after intracranial interleukin-1β injection
Source: J Neuroinflammation. 2014 Oct 17;11:178. doi: 10.1186/s12974-014-0178-3 (PMC4201684; doi:10.1186/s12974-014-0178-3)
Supplement: Additional file 2: Figure S1. — Viral pre-conditioning selectively increases chemokine expression in the brain 12 hours following the microinjection of IL-1β. The microinjection of IL-1β into the brain caused changes in absolute gene copies of mRNA for (A) CXCL-1; (B) CCL-2; (C) CXCL-10; (D) IL-1β; (E) CCL-3 and (F) CCL-4. Intravenous injections are indicated by italic text. Results are expressed as mean ± SEM (n =3), *P <0.05. [file 12974_2014_178_MOESM2_ESM.pptx]

## Slide 1
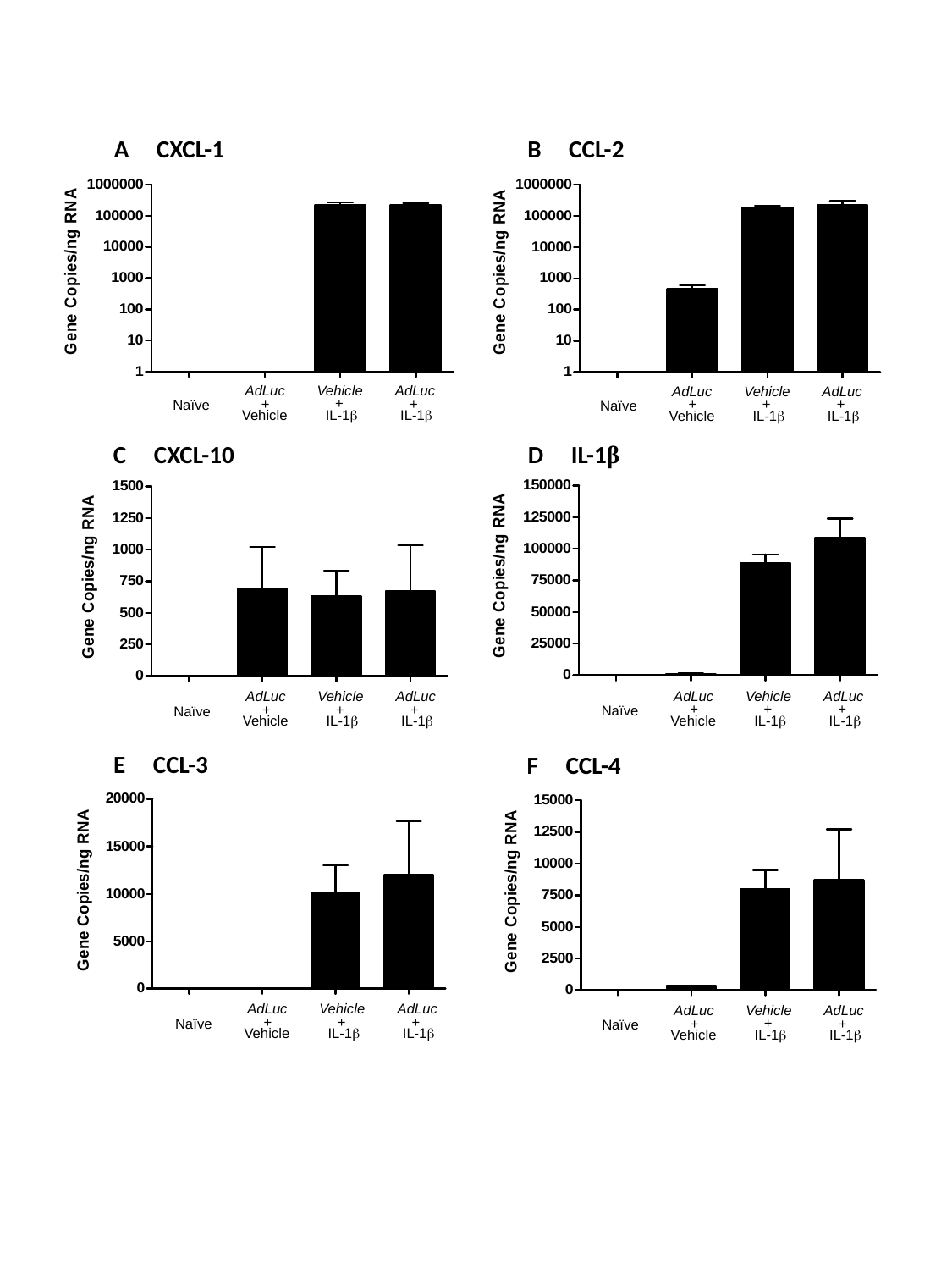

B CCL-2
A CXCL-1
AdLuc
Vehicle
AdLuc
+
+
+
Naïve
Vehicle
IL-1β
IL-1β
AdLuc
Vehicle
AdLuc
+
+
+
Naïve
Vehicle
IL-1β
IL-1β
C CXCL-10
D IL-1β
AdLuc
Vehicle
AdLuc
+
+
+
Naïve
Vehicle
IL-1β
IL-1β
AdLuc
Vehicle
AdLuc
+
+
+
Naïve
Vehicle
IL-1β
IL-1β
E CCL-3
F CCL-4
AdLuc
Vehicle
AdLuc
+
+
+
Naïve
Vehicle
IL-1β
IL-1β
AdLuc
Vehicle
AdLuc
+
+
+
Naïve
Vehicle
IL-1β
IL-1β
