# Supplementary figures and images for: Viral pre-challenge increases central nervous system inflammation after intracranial interleukin-1β injection
Source: J Neuroinflammation. 2014 Oct 17;11:178. doi: 10.1186/s12974-014-0178-3 (PMC4201684; doi:10.1186/s12974-014-0178-3)

**A CXCL-1**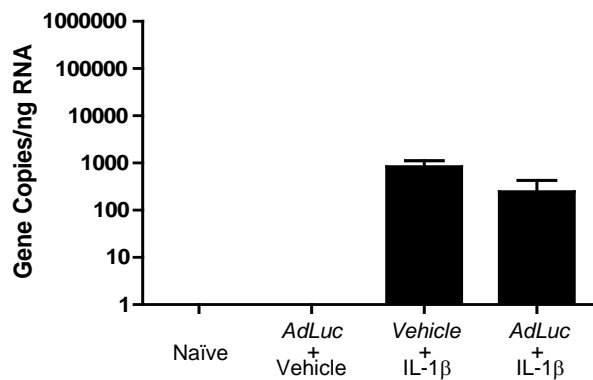**B CCL-2**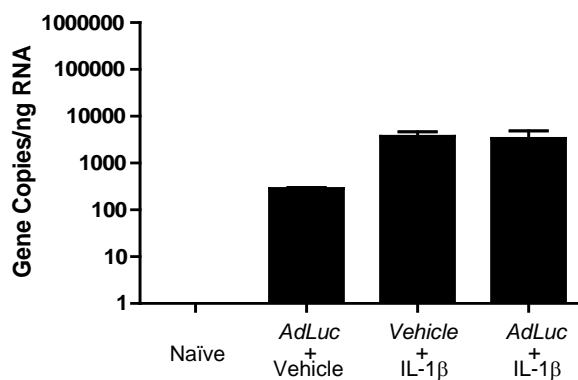**C CXCL-10**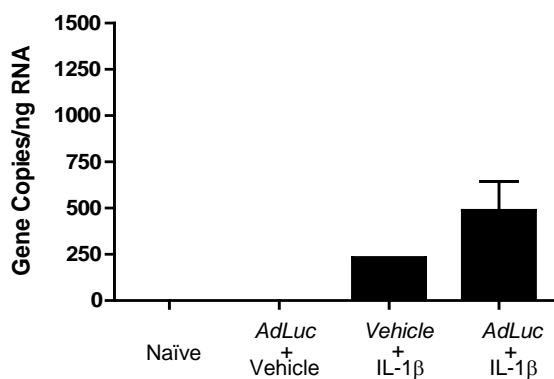**D IL-1β**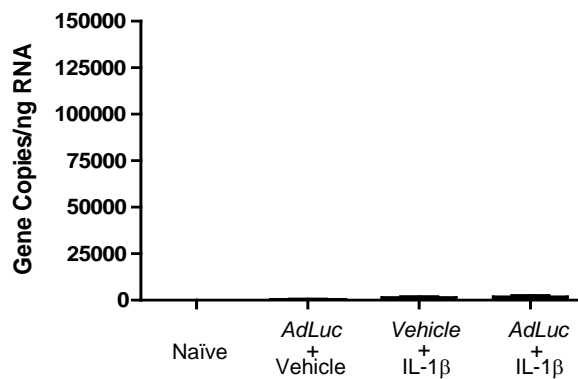**E CCL-3**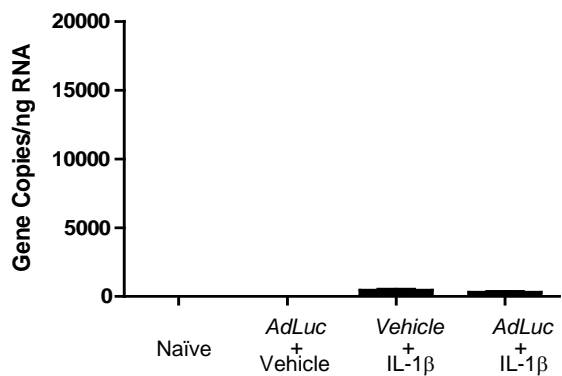**F CCL-4**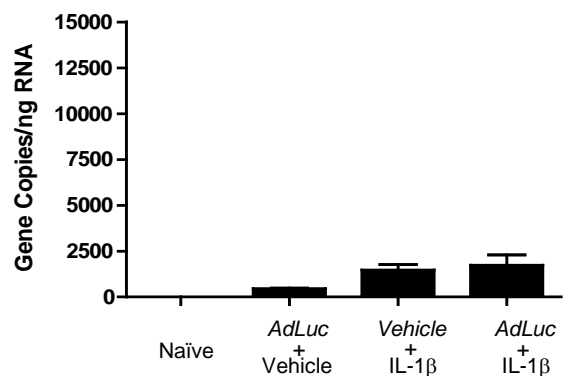

Supplement: Additional file 3: Figure S2. — Viral pre-conditioning selectively increases chemokine expression in the brain 7 days following the microinjection of IL-1β. The microinjection of IL-1β into the brain caused changes in absolute gene copies of mRNA for (A) CXCL-1; (B) CCL-2; (C) CXCL-10; (D) IL-1β; (E) CCL-3 and (F) CCL-4. Intravenous injections are indicated by italic text. Results are expressed as mean ± SEM (n =3), *P < 0.05. [file 12974_2014_178_MOESM3_ESM.pdf]

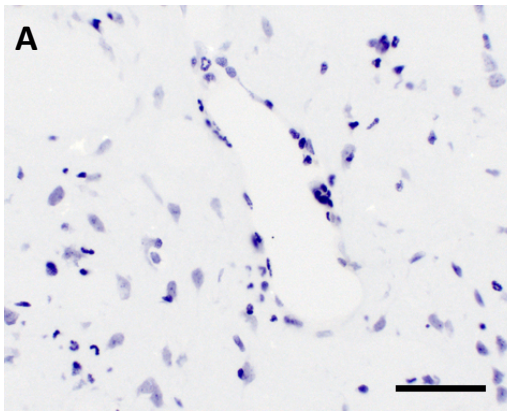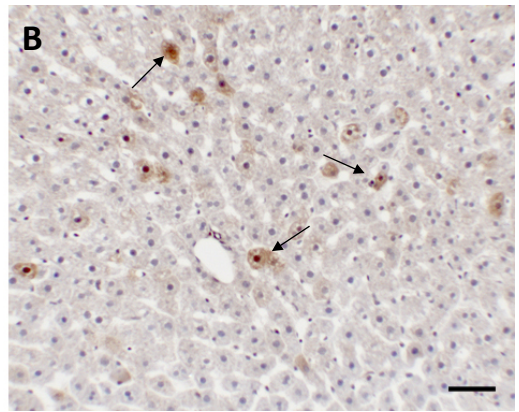

Supplement: Additional file 4: Figure S3. — AdLuc administered intravenously does not enter the brain. Micrographs illustrate representative sections of (A) brain, and (B) liver of animals administered AdLuc intravenously. In all treatment groups receiving intravenous AdLuc, no immuno-labelling for luciferase was observed in the brain at any time point, despite the microinjection of vehicle or IL-1β into the brain, but was clearly observed in the liver (B) of the same animals. Immuno-positivity for luciferase shown in brown (arrows). Scale bars represent 50 μm. [file 12974_2014_178_MOESM4_ESM.pdf]

### A CCL-3

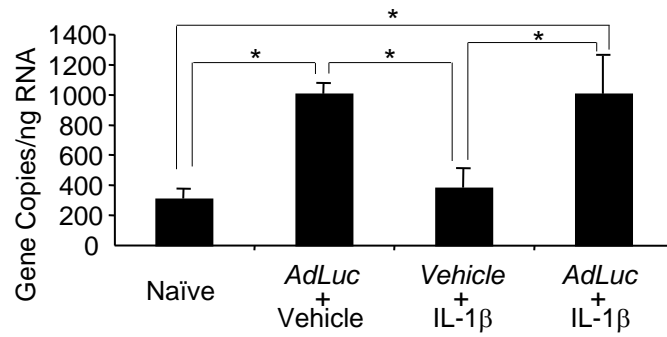

### B CCL-4

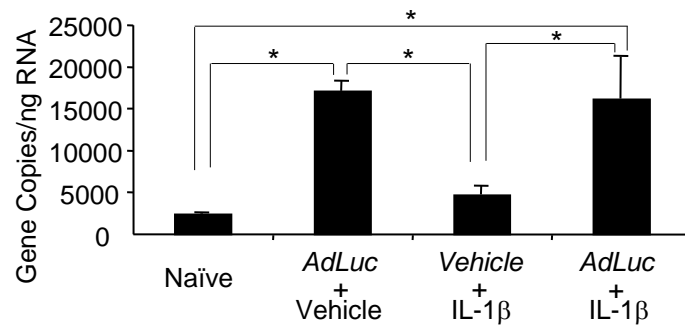

Supplement: Additional file 5: Figure S4. — Viral pre-conditioning causes selective up-regulation of macrophage-specific chemokines in the liver, irrespective of the challenge to the brain. The microinjection of IL-1β into the brain did not alter naïve chemokine mRNA levels in the liver 3 days later. A pre-challenge with AdLuc caused significant increases in both (A) CCL-3 and (B) CCL-4, irrespective of the challenge into the brain. Intravenous injections are indicated by italic text. Results are expressed as mean ± SEM (n =3), *P <0.05. [file 12974_2014_178_MOESM5_ESM.pdf]
